# Soluble Serum αKlotho Is a Potential Predictive Marker of Disease Progression in Clear Cell Renal Cell Carcinoma

**DOI:** 10.1097/MD.0000000000001917

**Published:** 2015-11-13

**Authors:** Margherita Gigante, Giuseppe Lucarelli, Chiara Divella, Giuseppe Stefano Netti, Paola Pontrelli, Cesira Cafiero, Giuseppe Grandaliano, Giuseppe Castellano, Monica Rutigliano, Giovanni Stallone, Carlo Bettocchi, Pasquale Ditonno, Loreto Gesualdo, Michele Battaglia, Elena Ranieri

**Affiliations:** From the Department of Emergency and Organ Transplantation – Nephrology, Dialysis and Transplantation Unit (MG, CD, CC, PP, GC, LG); Department of Emergency and Organ Transplantation, Urology, Andrology and Kidney Transplantation Unit, University of Bari, Bari (GL, MR, CB, PD, MB); Department of Medical and Surgical Sciences, Clinical Pathology, Nephrology, Dialysis and Transplantation Unit, University of Foggia, Foggia, Italy (GG, GS, ER).

## Abstract

Renal cell carcinoma (RCC) accounts for approximately 3% of adult malignancies, and clear cell RCC (ccRCC), that has a high metastatic index and high relapse rate, is the most common histological subtype. The identification of new biomarkers in ccRCC is fundamental for stratifying patients into prognostic risk groups and to guide therapy. The renoprotective antiaging gene, αKlotho, has recently been found to work as a tumor suppressor in different human cancers. Here, we evaluated αKlotho expression in tissue and serum of ccRCC patients and correlated it with disease progression. Tissue αKlotho expression was studied by quantitative RT-PCR and immunohistochemistry. In addition, soluble serum αKlotho levels were preoperatively measured in 160 patients who underwent nephrectomy for RCC with ELISA. Estimates of cancer-specific (CSS) and progression-free survival (PFS) were calculated according to the Kaplan–Meier method. Multivariate analysis was performed to identify the most significant variables for predicting CSS and PFS. αKlotho protein levels were significantly decreased in RCC tissues compared with normal tissues (*P* < 0.01) and the more advanced the disease, the more evident the down-regulation. This trend was also observed in serum samples. Statistically significant differences resulted between serum αKlotho levels and tumor size (*P* = 0.003), Fuhrman grade (*P* = 0.007), and clinical stage (*P* = 0.0004). CSS and PFS were significantly shorter in patients with lower levels of αKlotho (*P* < 0.0001 and *P* = 0.0004, respectively). At multivariate analysis low serum levels of αKlotho were independent adverse prognostic factors for CSS (HR = 2.11; *P* = 0.03) and PFS (HR = 2.18; *P* = 0.03).

These results indicate that a decreased αKlotho expression is correlated with RCC progression, and suggest a key role of declining αKlotho in the onset of cancer metastasis.

## INTRODUCTION

The anti-aging protein, αKlotho, is expressed in several organs, such as the parathyroid glands, choroid plexus of the brain and predominantly in the distal tubular epithelial cells of the kidney.^1,2^ Full-length αKlotho is a single-pass transmembrane protein that exists in 2 forms, membrane and secreted αKlotho, that exert different functions.^3^ The membrane form acts as coreceptor for fibroblast growth factor-23 (FGF-23) while the soluble form of αKlotho (sKlotho) can be cleaved by ADAM 10/17, proteases anchored to the membrane. When sKlotho is released into the circulation, it works as a humoral factor exerting different biological effects and works independently of FGF-23 signaling. The kidney is the major source of serum αKlotho in humans and, as recently described, this organ is involved in αKlotho homeostasis, responsible for producing and releasing αKlotho into the circulation and in clearing αKlotho from the blood into the urinary lumen.^4^ A αKlotho deficiency results in severe growth retardation, age-related disorders, and premature death.^5^ This protein is responsible for many pleiotropic actions such as tissue protection from oxidative stress, fibrosis, apoptotic stimuli but also for regulating blood phosphate and vitamin D3 levels and the activity of multiple cells surface calcium and potassium ion channels.^6–9^ Furthermore, numerous studies support a contribution of αKlotho to the regenerative response and stem cells preservation in neurodegenerative diseases and hematopoiesis.^10^ Recent findings show that an αKlotho deficiency contributes to kidney dysfunction and chronic kidney disease (CKD) progression, while the restoration of this protein attenuates CKD progression and reduces kidney parenchyma tissue injury.^11,12^ Recently, the role of αKlotho has been more closely investigated as tumor suppressor in several types of human cancers such as cervical, lung, colorectal, hepatocellular gastric, breast and pancreatic cancer.^13^ The secreted form of αKlotho acts as a autocrine or paracrine factor to regulate the activities of ion channels and to inhibit critical signaling pathways in cancer.^14^ In particular, the soluble αKlotho binds to Wnt ligands preventing its hyperactivation and subsequent aberrant cell proliferation and differentiation.^15^ αKlotho also regulates the insulin/insulin-like growth factor-1 (IGF-1) pathway by reducing IGF-1 receptor that is involved in tumor development, autophagy, and resistance to chemotherapies.^13^ Furthermore, the anti-aging protein reduces cellular senescence by repressing the p53/p21 pathway and suppresses the epithelial-to-mesenchymal transition by inhibiting TGF-β1 signaling.^16,17^ The loss of αKlotho gene expression has been reported to be mainly associated with the hypermethylation promoter DNA and histone deacetylation in human hepatocellular, gastric, colorectal, and cervical cancer representing predictive factors for the poor prognosis of these tumors.^18–20^ Although an aberrant expression of tissue αKlotho protein has been reported in a number of cancers including Renal Cell Carcinoma (RCC),^21^ the issue as to whether serum αKlotho could be a useful biomarker for RCC is still controversial. RCC is the predominant renal malignancy, and clear cell RCC (ccRCC) is the most common histological subtype.^22,23^ RCC is a highly aggressive tumor entity since the symptoms caused by this cancer normally appear at a relatively late stage of the disease, explaining why approximately 30% of patients are at an advanced stage of the disease at the time of diagnosis. Therefore, there is a crucial need to identify specific molecular biomarkers at the time of nephrectomy serving to predict a potential RCC progression.^24^ A prognostic role has been proposed for several circulating biomarkers associated with different features of RCC biology, including carbonic anhydrase IX (CAIX), hypoxia-inducible factor-1α (HIF1α), CA15-3, and some cancer metabolism-related proteins.^25–28^

An early identification of metastasis risk could foster a more precise prediction of clinical outcomes, and the stratification of RCC patients into subsets that could benefit from specific targeted therapies. Aim of this study was to investigate the circulating and tissue expression of αKlotho in RCC patients in order to evaluate whether circulating αKlotho may be a prognostic marker for RCC progression.

## MATERIAL AND METHODS

### Study Population

RCC tissue samples were obtained from a total of 24 patients. Immediately after surgery, tumor (T) and paired adjacent nontumoral renal parenchyma, denominated “nontumoral renal tissue” (NT), were frozen at −80°C according to a standard procedure and stored. Inclusion criteria were histologically confirmed ccRCC and no previous preoperative therapy. qRT-PCR was performed on a total of 36 tissues, consisting of 18 paired ccRCC and NT kidney tissues; immunohistochemistry was performed on a total of 20 ccRCC renal biopsies, and results were compared with those of their respective normal kidney portion.

Serum αKlotho was preoperatively measured in a cohort of 160 patients who underwent radical or partial nephrectomy for clear cell RCC at our Institution between June 2007 and December 2014, and in 20 healthy adult volunteers with no evidence of malignancy. The study was approved by the local Ethical Committee and informed consent was obtained from all patients according to the Declaration of Helsinki. Patients with an estimated glomerular filtration rate (eGFR calculated using MDRD equation) <60 mL/min/1.73 m^2^ were excluded from the study. In addition, patients with metabolic diseases (such as diabetes mellitus), with abnormal values of C-reactive protein or erythrocyte sedimentation rate, and with other signs of systemic inflammation, were excluded. All patients were preoperatively staged by thoraco-abdominal computed tomography (CT) or magnetic resonance imaging. Pretreatment clinical stage was assigned according to the 7th edition of the AJCC-UICC TNM classification.^25^ The 2004 World Health Organization and Fuhrman classifications were used to attribute histological type and nuclear grade, respectively. After treatment, patients were followed up according to the European Association of Urology guidelines.

### qRT-PCR

Collected samples were processed for total RNA extraction from 50 to 100 mg of fresh frozen tissue using the TRIzol reagent (Invitrogen, Life Technologies). RNA samples were purified using the RNeasy Mini kit (Qiagen, Hilden, Germany) and quantified using the NanoDropTM 1000 Spectrophotometer (NanoDrop Technologies, Berlin, Germany). RNA quality was determined by running aliquots on the 2100 Bioanalyzer (Agilent Technologies, Waldbronn, Germany). 200 ng of total RNA extracted from 18 ccRCC tumor sample (T) and 18 their matched nontumor (NT) was used in a 10 μL reverse transcription (RT) reaction using the High Capacity cDNA Reverse Transcription Kit (Applied Biosystems, Foster City, CA), following the manufacturer's instructions. One microliter of each undiluted cDNA in triplicate was used to perform quantitative real-time PCR amplification on the Biorad CFX 96 Instrument using the following Taqman Gene Expression Assays (Applied Biosystems, Foster City, CA): Hs00183100_m1(Klotho); Hs03003631_g1(18S RNA). No template controls were included as negative controls for each TaqMan assay. Amplification parameters were as follows: hot start at 95°C for 10 min; 40 amplification cycles (95°C for 15 s, 60°C for 1 min). Klotho fold change expression was then calculated according to the 2^−ΔΔCT^ method.^29^ The mean delta Cq, previously known as the threshold cycle (Ct), of the biological replicates within the nontumor group was considered calibrator. The significance of the differential expression was tested by *t* test, using a *P* value cutoff of 0.05.

### αKlotho Immunohistochemistry (IHC)

αKlotho staining was performed on human paraffin-embedded renal sections with rabbit polyclonal anti-Klotho 1:200 antibody (Lifespan Biosciences, Seattle, WA). After rehydration and antigenic retrieval, 2-μm-thick sections were incubated for 10 min with H_2_O_2_ (3%) and then for 5 min with tween-20 (0.1%). The sections were blocked with serum-free protein block (Dako, Glostrup, Denmark) for 10 min at room temperature and then incubated with the primary antibody. Immune complexes were detected by the Peroxidase/DAB Dako Real EnVision Detection System, according to the manufacturer's instructions (Dako). The peroxidase reaction was shown by a brown precipitate, counterstained with Mayer's hematoxylin (blue) and mounted with glycerol (DakoCytomation, Carpintera, CA). Negative controls were prepared using rabbit IgG, polyclonal – isotype control antibody (Abcam, Cambridge, UK). Digital images were obtained using the Aperio ScanScope CS2 device (Aperio Technologies, Vista, CA) and further analyses of the scanned images were performed with the ImageScope V12.1.0.5029 (Aperio). Specific staining was quantified by applying the Positive Pixel Count v9_v10.0.0.1805 algorithm (Aperio) and expressed as percentage of positive pixels in the analyzed area.^30^

### Microarray Data Analysis

Twenty total samples were used for exon array analysis, 10 ccRCC tumor sample (T) and their matched nontumor (NT) kidney tissues samples, as previously described.^31^ Exon array data are deposited in GEO with Series accession number GSE47032. Exon array data were analyzed using GeneSpring GX 11.5 statistical analysis software. Gene expression values were log transformed and differentially expressed genes were identified based on a fold-change greater than 2 and *P* value less than 0.05, according to the comparison of the 2 groups by a paired *t* test.

### Statistical Analysis

Statistical calculations were performed with MedCalc 9.2.0.1 (MedCalc software, Mariakerke, Belgium) and PASW 18 software (PASW 18, SPSS Inc, Chicago, IL). Comparisons of Klotho median values between different groups were evaluated by Mann–Whitney *U* test. Receiver-operating characteristic (ROC) curve analysis was performed to identify the αKlotho cutoffs for survival stratification and to compare the prognostic performance of serum αKlotho versus CA 15-3 tumor marker. Cancer-specific survival (CSS) was measured from the date of surgery to the date of death from RCC. Patients lost to follow-up or who died of RCC-unrelated causes were censored. Progression-free survival (PFS) was calculated from the date of surgery to the date of disease recurrence. CSS and PFS estimates were calculated according to the Kaplan–Meier method and compared with the log-rank test. Spearman's correlation was applied to evaluate associations between αKlotho expression and tumor size, grade, and stage. Univariate and multivariate analyses were performed using the Cox proportional hazards regression model to identify the most significant variables for predicting CSS and PFS. A backward selection procedure with removal criterion *P* >0.10 based on likelihood ratio tests was performed. *P* values <0.05 were considered statistically significant.

Immunohistochemical data were presented as mean ± standard deviation (SD). Statistical analysis was performed using GraphPad Prism 5 (GraphPad Software, La Jolla, CA). Paired, unpaired Student *t* test, Mann–Whitney test, or ANOVA were used as appropriate. A *P* value <0.05 was considered statistically significant.

## RESULTS

### αKlotho Expression in RCC Cancer

In a previous study we performed a genome-wide analysis on RCC patients with no evidence of other diseases at the time of diagnosis, comparing T versus paired adjacent NT renal tissue of the same patient, as described in the “Methods” section.^31^ We identified 4 novel ccRCC-associated biomarkers, PTP4A3, LAMA4, KCNJ1, and TCF21, whose sensitivity ranged from 77.8% to 100%. Given the emerging importance of αKlotho as a tumor suppressor in different kinds of tumor, we explored our database to assess whether the αKlotho gene was modulated in the previously analyzed RCC patients. We found a modulated αKlotho gene expression in our data set (absolute FC = −41.97; Log FC = −2.069; *P* = 0.0007), whereby the gene expression was downregulated in tumor tissue compared with NT. To support these data we analyzed the differential expression of αKlotho mRNA between RCC tissues and normal renal tissues by data-mining of 6 independent Oncomine microarray gene expression datasets (Table 1).^32–37^ We found that αKlotho expression was significantly down-regulated in all 6 datasets, with a median fold change of −3.954.

**TABLE 1:**
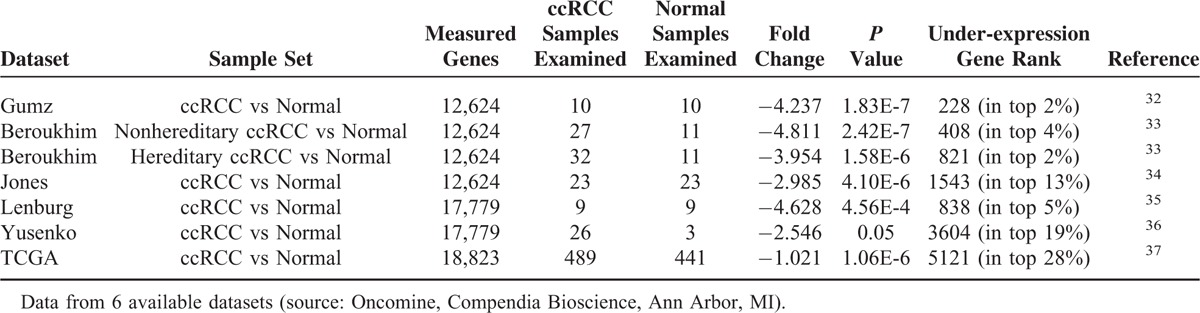
Klotho Expression in ccRCC

**FIGURE 1:**
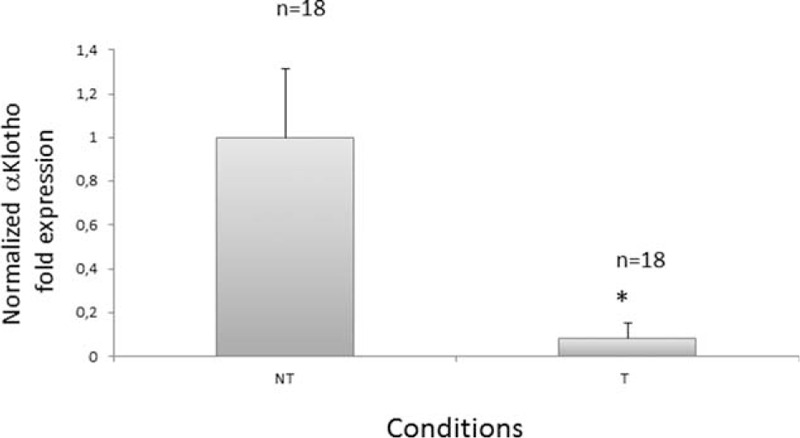
Klotho gene expression levels evaluated by qRT–PCR. Histogram represents mean ± SD of normalized Klotho gene expression levels by quantitative real-time PCR in 18 ccRCC samples (T) compared with their matched-NT (∗*P* < 0.001).

To confirm the above findings, we first performed quantitative qRT-PCR and analyzed αKlotho mRNA levels in 18 RCC patients, comparing T versus paired adjacent NT renal parenchyma. αKlotho levels are presented in Figure 1. Normalized gene expression levels for αKlotho were significantly lower in the ccRCC compared with the corresponding matched NT kidney tissue.

**FIGURE 2:**
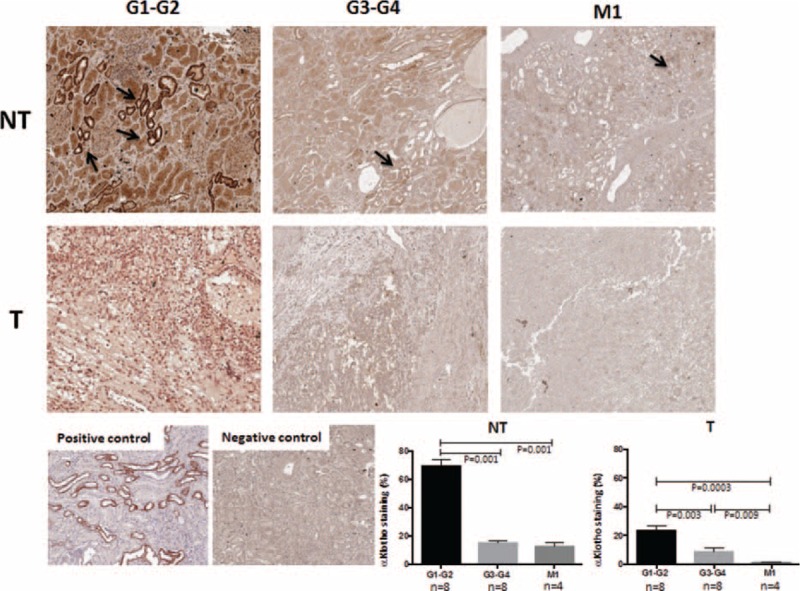
In vivo analysis for Klotho expression using immunohistochemistry. Renal Klotho expression was evaluated in RCC tumor tissues (T) and matched nontumoral (NT) kidney portions from patients with different grades of RCC (G1–G2 and G3–G4) and patients with metastases (M1). Klotho was expressed at tubular level in NT portion of G1–G2 RCC patients and significantly decreased in G3–G4 and M1 patients. In T tissue, αKlotho was slightly expressed in patients with G1–G2 and patients with G3–G4 and M1 showed a very low or undetectable expression. Klotho staining was quantified as described in the “Methods” section and was expressed as mean ± SD. All images are from a single patient and are representative of all 8 patients for G1–G2 and G3–G4 groups and 4 patients for M1. The positive control is a normal kidney tissue and negative control is obtained as described in the “Methods” section.

We also conducted IHC analysis and examined αKlotho expression in tissue from 20 RCC patients, observing a similar protein and mRNA expression pattern (Fig. 2). However, we observed an association between αKlotho expression levels and differentiation status, disease grade, and presence of metastases. We observed that renal tubular αKlotho was expressed in the NT tissue of G1–G2 patients and significantly decreased with the progression of the disease, in G3–G4 patients (*P* = 0.0001), as well as in metastatic patients (M1, *P* = 0.0001). In the T tissue, we found a dramatically lowered αKlotho expression that was detectable only in RCC patients with G1–G2. In advanced RCC (G3–G4) as well as M1 patients, αKlotho expression significantly decreased when compared with early stage of disease (*P* = 0.0032 and *P* = 0.0003 respectively).

### Serum Klotho in RCC Patients

**FIGURE 3:**
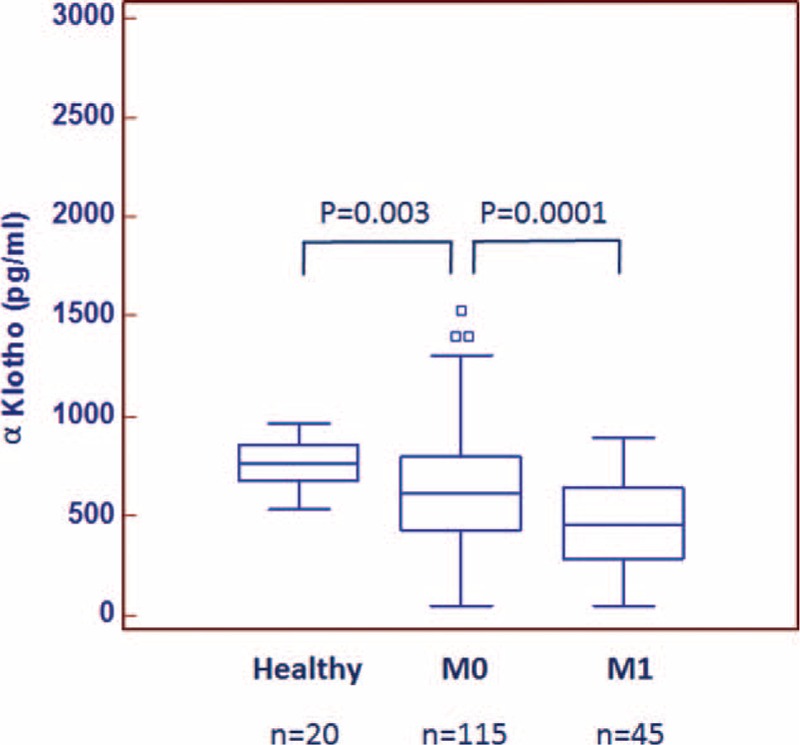
Median αKlotho levels in 20 healthy subjects compared with 45 patients with metastatic (M1) and 115 nonmetastatic (M0) ccRCC.

**FIGURE 4:**
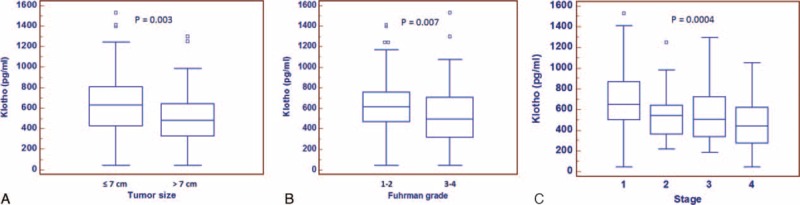
Serum αKlotho levels stratified according to tumor size (≤7 cm vs >7 cm; panel A) and nuclear grade (Fuhrman 1–2 vs 3–4, panel B).

We next examined the soluble form of αKlotho in a cohort of 160 RCC patients. Detailed clinical and pathological characteristics of the patients are summarized in Table 2. Median age at diagnosis was 61 years (range: 26–85); median tumor size was 6.0 cm (range: 1–24 cm). Median αKlotho serum levels were significantly lower in RCC patients (nonmetastatic = 609.5 pg/mL; metastatic = 459.5 pg/mL) than in healthy subjects (763 pg/mL) (Fig. 3). Statistically significant differences resulted between αKlotho values and tumor size (≤7 vs >7 cm; 630.7 vs 484.5 pg/mL; *P* = 0.003), Fuhrman grade (≤2 vs >2; 619.5 vs 484.3 pg/mL; *P* = 0.007) and clinical stage (Stage I = 653.2; II = 543.2; III = 505.1; IV = 442 pg/mL; *P* = 0.0004) (Fig. 4, panel A–C). These results were confirmed by Spearman's correlation. In particular, an inverse correlation was observed comparing αKlotho levels and tumor size (rs = −0.23, *P* = 0.005), grade (rs = −0.25, *P* = 0.002), and stage (rs = −0.33, *P* = 0.0001).

**TABLE 2:**
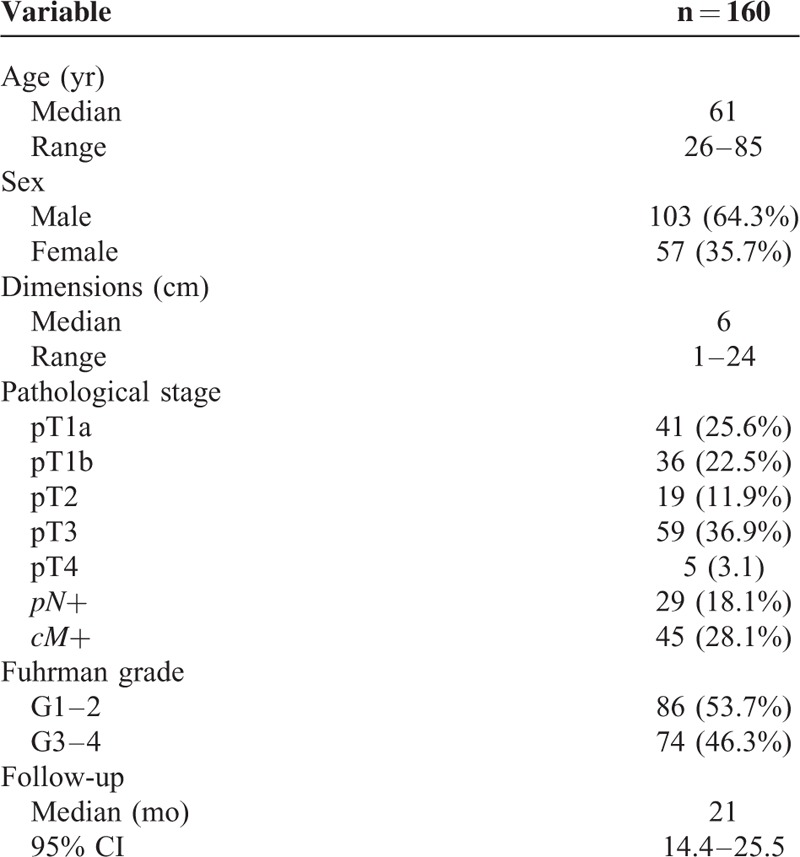
Clinical and Pathological Characteristics of RCC Patients

**FIGURE 5:**
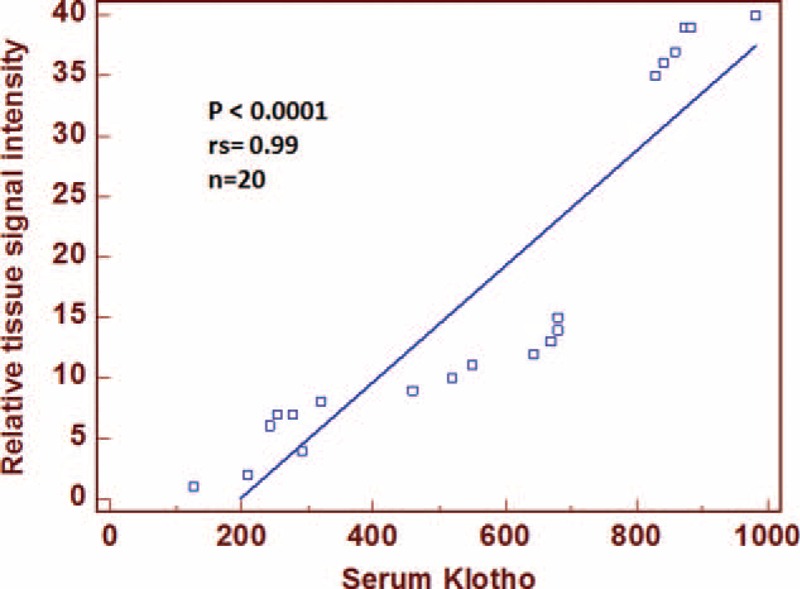
Spearmann correlation coefficient (rs) and linear regression line between αKlotho tissue signal intensity and serum values from 20 ccRCC patients.

To evaluate the association between the total amount of Klotho in RCC tissue and the soluble form of αKlotho, we performed a correlation between the tissue signal intensity and the serum values from 20 RCC patients. Spearman's test showed a significant direct correlation between tissue and serum αKlotho levels (rs = 0.99, *P* < 0.0001) (Fig. 5).

**FIGURE 6:**
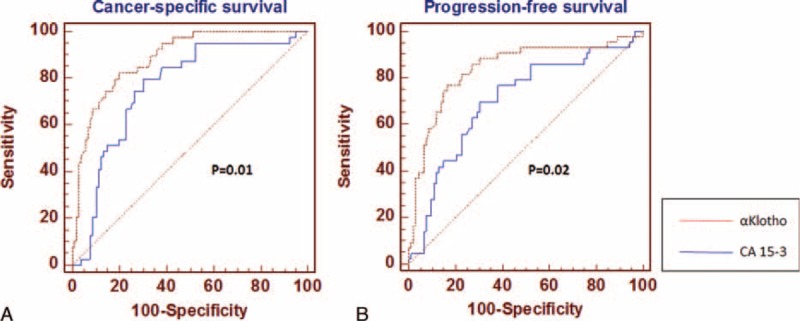
Comparison of αKlotho and CA 15-3 receiver-operating characteristic (ROC) curves for cancer-specific survival (panel A), and progression-free survival (panel B).

ROC curve analysis (Fig. 6) showed that αKlotho had the best predictive values for RCC-specific mortality and progression (AUC = 0.885 and AUC = 0.838, respectively), outperforming CA 15-3 serum levels (AUC = 0.759 and AUC = 0.703, respectively).

Sensitivity and specificity of serum αKlotho for CSS and PFS are shown in Table 3.

**TABLE 3:**
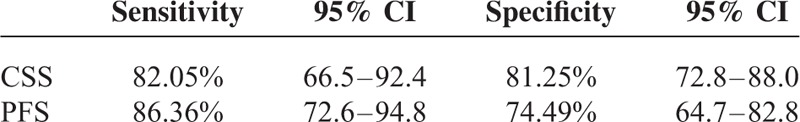
Sensitivity and Specificity of αKlotho for Cancer-Specific Survival (CSS) and Progression-Free Survival (PFS)

### Cancer-Specific Survival

To evaluate the association between patients survival and αKlotho serum levels, we classified the entire population by high versus low expression levels according to the cutoff provided by receiver-operating characteristic (ROC) curve analysis. A cutoff of 400 pg/mL provided the optimal balance between sensitivity (46%) and specificity (82%).

**FIGURE 7:**
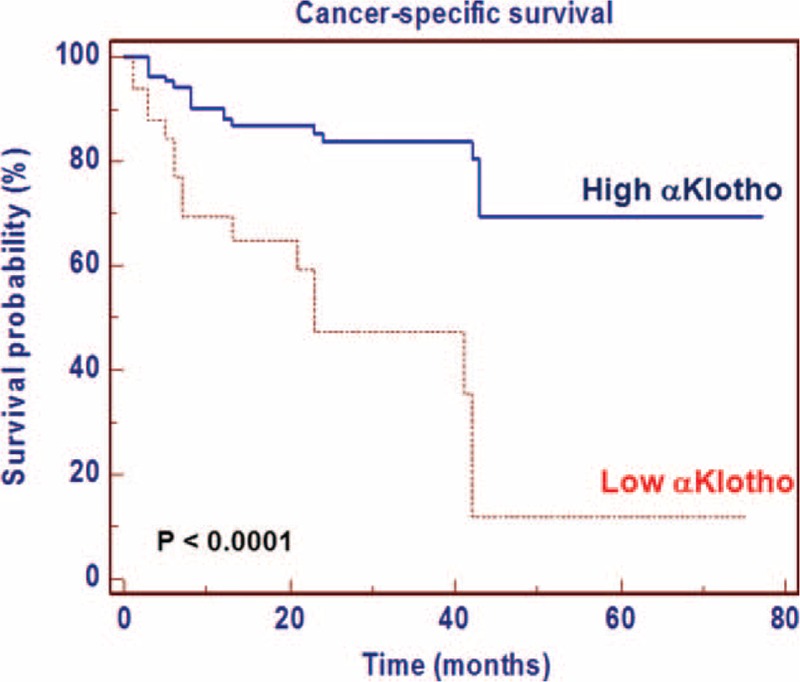
Kaplan–Meier cancer-specific survival (CSS) curves, stratified by αKlotho serum levels.

After a median follow-up of 21 months (95% CI: 14.4–25.5), 39 (24.4%) patients had died of RCC. Kaplan–Meier survival curves for CSS, stratified by αKlotho serum levels, are shown in Figure 7. CSS was significantly decreased for patients with low levels of αKlotho (*P* < 0.0001). Univariate analyses for the predefined variables showed that age, tumor size, pathological stage, presence of visceral metastases, TNM stage, Fuhrman grade, and low αKlotho serum levels were significantly associated with the risk of death (Table 4). At multivariate analysis by Cox regression modeling, tumor size, the presence of visceral metastases, high Fuhrman grade, and low αKlotho serum levels were independent adverse prognostic factors for CSS (Table 4).

**TABLE 4:**
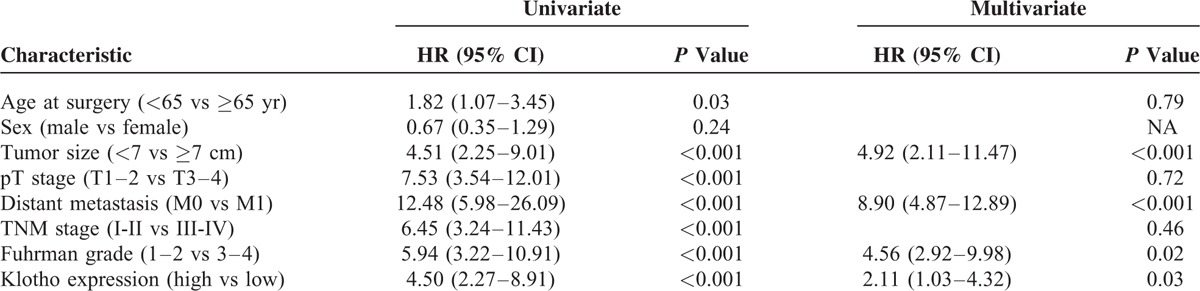
Univariate and Multivariate Analyses for Cancer-Specific Survival

### Progression-Free Survival

**FIGURE 8:**
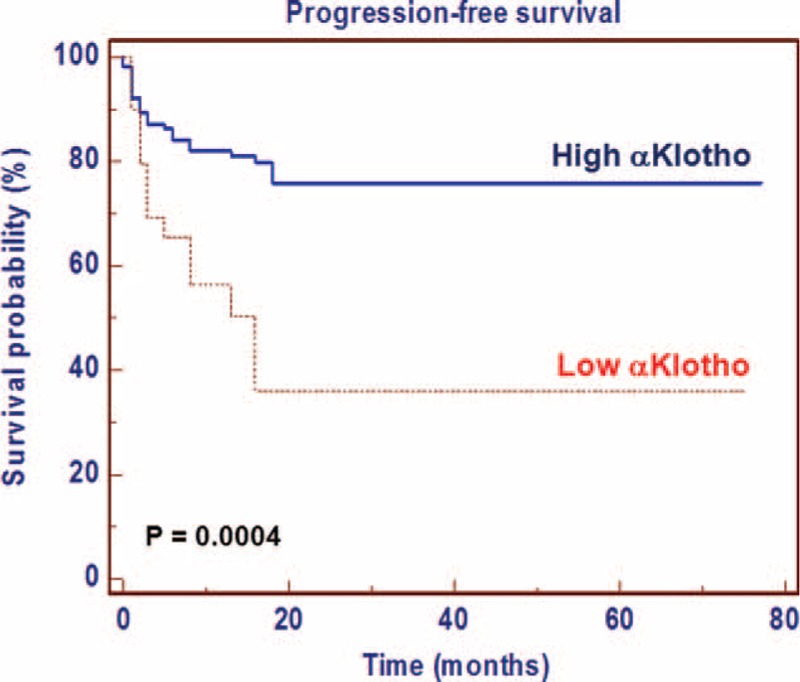
Kaplan–Meier progression-free survival (PFS) curves, stratified by αKlotho serum levels.

After surgery, 44 (27.5%) patients showed disease progression, after a median PFS of 17 months (95% CI: 13–23). Kaplan–Meier survival curves for PFS, stratified by αKlotho serum levels, are shown in Figure 8. PFS was significantly decreased for patients with low levels of αKlotho (*P* = 0.0004). Univariate analyses for the predefined variables showed that tumor size, pathological stage, presence of visceral metastases, TNM stage, Fuhrman grade, and low levels of αKlotho were significantly associated with the risk of death (all *P* = 0.0001). At multivariate analysis, only pT stage, presence of visceral metastases, and low levels of αKlotho were independent adverse prognostic factors for PFS (Table 5).

**TABLE 5:**
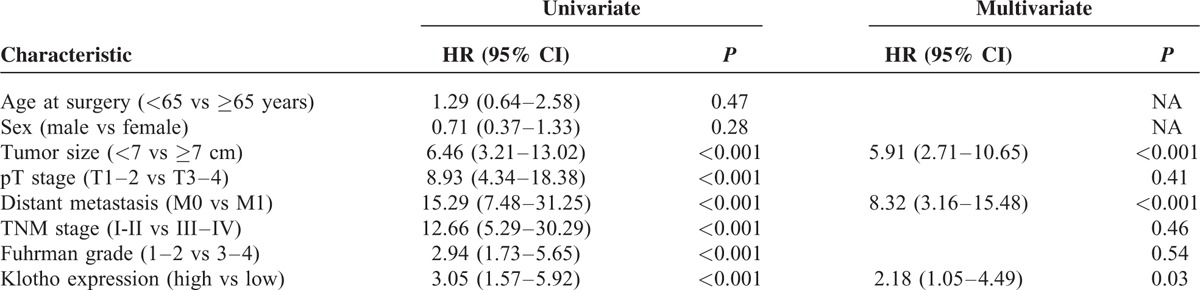
Univariate and Multivariate Analyses for Progression-Free Survival

## DISCUSSION

In the present study, we evaluated the clinical significance of soluble αKlotho in RCC survival and progression. The currently available clinical biomarkers of RCC show poor diagnostic sensitivity, and are also very poor predictors of metastasis. Thus, the identification of molecular biomarkers for RCC patients remains an ambitious challenge in oncologic research. Over the past decade, rapid advances in high-throughput technologies have offered new opportunities for biomarker discovery. In our previous study, we performed a genome-wide gene expression analysis to identify differentially expressed genes in ccRCC tumoral tissue compared with adjacent nontumoral renal tissue from the same patient.^31^ We reported 4 novel RCC associated biomarkers, PTP4A3, LAMA4, KCNJ1, and TCF21 genes, showing high sensitivity. Among the differentially expressed genes, we found that the αKlotho gene was highly modulated in our data set. Given its relevant role in kidney homeostasis as well as its function as a tumor suppressor,^12,13^ here we focused our attention on αKlotho gene and protein expression level. αKlotho is an early biomarker of acute kidney injury^11^ and as our group has recently reported, activation of the complement system is one of the main mechanisms leading to the down-regulation of αKlotho in renal injury.^37^ A reduced αKlotho mRNA expression in ccRCC was confirmed in 6 microarray gene expression datasets deposited in the Oncomine database (https://www.oncomine.org/resource/login.html), analyzing a total of 616 cancer tissue samples and 508 normal controls.^32–37^ In particular, αKlotho was downregulated in all tumor samples compared with normal tissue, and this result was in accordance with our findings.

Gene and protein expression was validated by qRT-PCR and immunohistochemistry, respectively. A significantly decreased αKlotho expression was found in ccRCC tissue compared with adjacent normal renal parenchyma. These findings are in accordance with other reports on different human tumors, including lung, breast, pancreatic, colon, cervical cancer, as well as in RCC, in which there was a very low transmembrane αKlotho expression. Interestingly, we observed that renal tubular αKlotho expression decreased with the progression of disease not only in the tumor tissue but also in the normal matched renal portion as compared with expression levels in early stage ccRCC patients. Recently, it has been reported that the kidney is the major source of circulating αKlotho in the systemic circulation, and is responsible for its clearance.^4^ On these premises, we evaluated, for the first time, the prognostic role of the soluble form of Klotho in sera of a large cohort of patients who underwent radical or partial nephrectomy for ccRCC. The main limitations of this study include the single-center nature of the report, retrospective nature, and single preoperative measurement of αKlotho rather than serial determinations.

Circulating αKlotho was measured in serum samples collected the day before surgery. Patients with all known clinical conditions that could cause abnormal values of circulating αKlotho—such as chronic kidney disease, metabolic diseases, and systemic inflammation—were excluded from the study. In our findings, serum αKlotho levels were higher in patients with low grade, low stage disease. Moreover, a significant inverse correlation resulted between αKlotho values and tumor size, grade, and stage. Kaplan–Meier survival curves stratified by αKlotho levels demonstrated that CSS was significantly decreased in patients with low serum values. Multivariate analysis showed that low levels of αKlotho, together with the presence of visceral metastases, Fuhrman grade ≥3, and tumor size ≥7 cm, were significantly predictive of death risk. In addition, this marker remained an independent prognosticator of outcome, in terms of PFS. Kaplan–Meier curves showed clear differences in PFS between patients with low versus high serum levels. The independent prognostic value of this protein for PFS was confirmed at multivariate analysis. These findings are in accordance with the results obtained by Zhu et al,^21^ which showed an association between intratumural αKlotho levels and RCC prognosis. In particular, in this study, αKlotho was immunohistochemically evaluated and its tissue expression was correlated with patients’ clinical characteristics. Patients with low tissue levels had a reduced overall survival compared with subjects with high tissue expression, and multivariate analysis confirmed that αKlotho tissue levels, in association with nuclear grade and primary tumor size, were independent prognostic factors. Our data corroborate the hypothesis that the reduced serum αKlotho levels in advanced RCC could be due to reduced renal synthesis. These results are in line with those recently observed in patients with chronic kidney disease (CKD) that showed a parallel reduction of renal αKlotho and serum αKlotho, correlated with disease progression and renal damage^38^

In a previous study, we showed that RCC patients with elevated serum levels of CA 15-3 had a reduced CSS and PFS, as compared with those with low CA 15-3 values.^26^ To evaluate to prognostic performance of serum αKlotho in predicting cancer-specific mortality and progression, we conducted a ROC curve analysis. ROC curve analysis shows a predictive superiority of αKlotho compared with CA 15-3 for both CSS and PFS, with high sensitivity and specificity.

In conclusion, we found that soluble αKlotho, as well as tissue levels (intratumoral αKlotho), are progressively reduced in patients with advanced ccRCC. Thus, sKlotho appears to be a novel, relevant biomarker reflecting the disease activity in patients with ccRCC. This opens new perspectives for a more effective disease monitoring, as well as for future therapeutic interventions.

## UNCITED REFERENCE

^38^.
